# Complete Chloroplast Genome Analysis of *Casearia kurzii*: Gene Loss at the IR Boundary and Monophyletic Evolution Within *Casearia*

**DOI:** 10.3390/plants14091356

**Published:** 2025-04-30

**Authors:** Kan Yan, Wandi Li, Chao Sun, Xin Lu, Xueqiong Zhou, Youyou Wang, Yongqiang Tian

**Affiliations:** 1 School of Biological and Pharmaceutical Engineering, Lanzhou Jiaotong University, Lanzhou 730070, China; liwandi2022@163.com (W.L.); fxq092424@163.com (X.Z.); idafnj@163.com (Y.W.); tianyq@mail.lzjtu.cn (Y.T.); 2 College of Agronomy, Gansu Agricultural University, Lanzhou 730070, China; sunc@gsau.edu.cn

**Keywords:** *Casearia kurzii*, comparative genomics, chloroplast genome, phylogenetic analysis, molecular markers

## Abstract

Genomic analysis is crucial for understanding the evolutionary history, phylogenetic relationships, and effective conservation of plant species. *Casearia kurzii* is an important medicinal plant of the genus *Casearia*, but its complete chloroplast genome has not previously been reported, limiting genetic studies and conservation efforts. In this study, we assembled and annotated the complete chloroplast genome of *C. kurzii* using Illumina sequencing technology and conducted a comparative genomics analysis with 14 closely related species to clarify its phylogenetic position within *Casearia*. The chloroplast genome was 157,998 bp, showing a typical quadripartite structure. Key findings included: (1) the loss of the *rpl22* gene at the IR boundary; (2) the identification of 60 simple sequence repeats (SSRs) and (3) the discovery of five candidate molecular markers for species-level identification. Phylogenetic analysis revealed that *C. kurzii* formed a strongly supported monophyletic clade (100% bootstrap support) with *C. velutina*, *C. decandra*, and *C. glomerata*, this clade originated approximately 15.8 million years ago. This study provides molecular tools for accurate identification and conservation of *C. kurzii* and related species, laying the foundation for exploring adaptive evolution within *Casearia* and advancing comparative genomics research.

## 1. Introduction

Chloroplasts are essential organelles in plant cells responsible for photosynthesis and biosynthesis [1]. They possess relatively independent genetic material and are semi-autonomous. The chloroplast genomes of most plants exhibit a typical tetrad circular structure, and their conserved gene composition, genomic structure, and GC content provide an ideal model for evolutionary research [2,3]. With the development of high-throughput sequencing technologies, the study of chloroplast genomes has demonstrated unique advantages in species identification, phylogeny, and population genetics due to their monophyletic inheritance and moderate evolutionary rates [4]. Previous studies, such as Liu et al.’s research on the chloroplast genomes of five species in the Hamamelidaceae family, have developed multiple molecular markers for intraspecific identification [5]; Jiang et al.’s comparative analysis of chloroplast genomes from 34 *Incarvillea* species revealed interspecific evolutionary relationships [6]; Nguyen et al. compared the chloroplast genomes of cultivated and wild *Baccaurea ramiflora* [7]. However, this important molecular resource has not been fully explored in ecologically and economically valuable species such as *Casearia kurzii* (Salicaceae).

**Figure 1:**
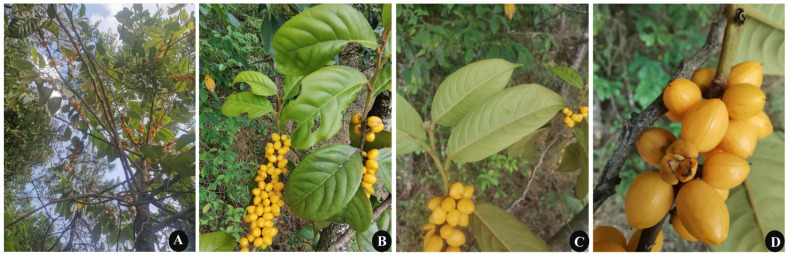
Morphological characteristics of *C. kurzii*. (**A**) Habitat in humid rainforest ecosystems, reaching 5–12 m in height. (**B**) Upper leaf surface membranous, lanceolate-oblong, smooth surface. (**C**) Lower leaf surface usually densely covered with yellow pubescence. (**D**) Mature fruits yellow elliptical drupes, 1.3–1.6 cm in length and 0.6–1.0 cm in diameter, with persistent calyx at base.

*C. kurzii* is a tropical plant distributed in Yunnan (China), northern Myanmar, and northeastern India [8,9]. In traditional medicine, its roots, stems, and leaves are widely used to treat conditions such as hypoglycemia, diarrhea, and constipation (Figure 1) [10,11,12]. Like other species in the genus *Casearia*, it occupies an important ecological niche in subtropical understory forests. However, its wild populations are increasingly threatened by habitat fragmentation due to deforestation and agricultural expansion [13]. Taxonomically, the genus *Casearia* (Table 1), which comprises over 200 pantropical species, presents significant challenges [14,15,16,17,18,19,20]. Ongoing taxonomic revisions, including the merging of formerly independent genera and the description of new species, have further complicated its systematics [21]. Morphological similarities, such as indistinct floral traits and overlapping leaf characteristics (e.g., short trichomes, size), complicate species identification. For example, newly discovered species from Madagascar (*Casearia anosyensis*, *Casearia montigena*) are mainly defined based on unstable leaf traits [22], while new neotropical groups like *Casearia grandiflora* face similar taxonomic challenges due to convergent morphology [23]. Despite efforts in molecular phylogenetic studies to resolve these issues, limited sampling and reliance on short plastid/nuclear markers (such as *rbcL* and *matK*) have led to ambiguous relationships [24]. This taxonomic uncertainty significantly hinders our understanding of the evolutionary history and ecological adaptation mechanisms of this genus. To date, only a few *Casearia* species, such as *C. grandiflora*, have undergone partial chloroplast microsatellite analysis [23], while complete chloroplast genomic studies on species like *Casearia glomerata* [25], *Casearia decandra*, and *Casearia velutina* [26] remain relatively preliminary. Moreover, detailed descriptions of chloroplast genome maps, as well as systematic assessments of simple sequence repeats (SSRs) and highly variable regions, are lacking. Notably, *C. kurzii* still lacks a complete chloroplast genome sequence, and no comprehensive molecular markers have been developed for this species. The absence of comparative chloroplast genome analyses across the genus has hindered the development of high-resolution markers, which are essential for evolutionary studies and conservation genetics.

To address these gaps, this study proposed two key hypotheses: (1) *C. kurzii* may possess unique structural features in its chloroplast genome; (2) high-resolution molecular markers can be developed from SSRs and hypervariable regions. This work represents the first complete sequencing, assembly, and analysis of the chloroplast genome of *C. kurzii*, coupled with a comparative genomic study involving 14 closely related species. The main objectives of this research were: (1) to characterize the complete chloroplast genome structure of *C. kurzii* and identify potential unique genomic features; (2) to detect SSRs and highly variable markers with taxonomic value and evaluate their efficacy in species discrimination within the genus; (3) to reconstruct phylogenetic relationships and estimate divergence times using single-copy genes, thereby helping to resolve taxonomic uncertainties; and (4) to provide molecular data to support the development of conservation strategies based on genetic diversity. This study offers new molecular insights into the taxonomy of *C. kurzii* and delivers practical tools for species identification and conservation genetics within *Casearia*. Ultimately, the results will enhance our understanding of the genus’s evolutionary history and support the science-based conservation of *C. kurzii*.

## 2. Results

### 2.1. Main Features of the Chloroplast Genome

**Figure 2:**
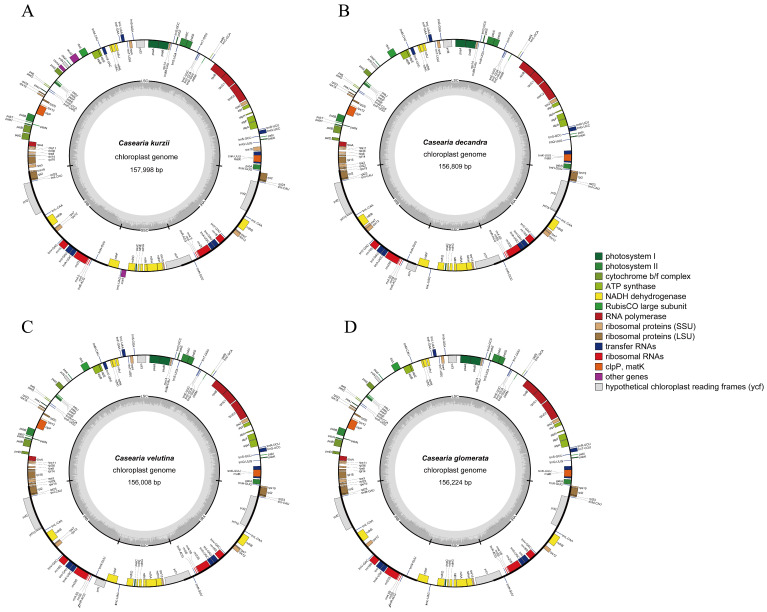
Comparative chloroplast genome maps of four *Casearia* species. (**A**) *Casearia kurzii*, representing the complete chloroplast genome sequenced and assembled in this study. (**B**) *Casearia decadra*, (**C**) *Casearia velutina*, and (**D**) *Casearia glomerata*, showing chloroplast genome maps generated based on data retrieved from [25,26], respectively. The inner circle indicates the range of the large single-copy (LSC), small single-copy (SSC), and inverted repeats (IRs). The darker gray area in the inner circle represents the GC content, while the lighter gray corresponds to AT content. The outer circle shows the genes at each locus, and transcribed clockwise genes are shown outside, while counterclockwise genes are inside.

In this study, the complete chloroplast genome of *C. kurzii* was sequenced and assembled. To compare the genomic characteristics of this species with related taxa, we further analyzed the chloroplast genome data of *C. velutina*, *C. decandra*, and *C. glomerata*, which were obtained from published sources (Figure 2 and Table 2). The total length of the *C. kurzii* chloroplast genome was 157,998 bp, and exhibited a typical quadripartite circular structure (Figure 2). This genome consisted of a large single-copy region (LSC, 85,806 bp) and a small single-copy region (SSC, 16,150 bp), separated by a pair of inverted repeats (IRa and IRb, each 28,021 bp). Based on the overall GC content analysis, the GC content of the *C. kurzii* chloroplast genome was 36.72%. The IR regions showed the highest GC content (42.05%), while the LSC and SSC regions had GC contents of 34.46% and 30.21%, respectively.

The chloroplast genome sizes of the four *Casearia* species were relatively similar, ranging from 156,008 bp (*C. velutina*) to 157,998 bp (*C. kurzii*) (Table 2). The lengths of their LSC, SSC, and IR regions also showed overall similarity. However, the LSC of *C. kurzii* was slightly longer, while the SSC of *C. velutina* was relatively longer. The overall GC content of the four species was also very close, ranging from 36.72% to 36.83%.

The chloroplast genome of *C. kurzii* encoded a total of 127 genes (counting duplicated genes in the IR regions twice), including 82 protein-coding genes, 37 tRNA genes, and 8 rRNA genes (for detailed inforation, see Table 3). Excluding the duplicated genes in the IR region (Table 2), the total number of unique genes for the four *Casearia* species was as follows: *C. kurzii* (110), *C. velutina* (110), *C. decandra* (110), and *C. glomerata* (107). This comparison revealed that *C. glomerata* had fewer unique genes, particularly in protein-coding genes (74 vs. 76) and tRNA genes (27 vs. 30), indicating that the variation in gene number was mainly associated with gene gains or losses near the IR boundaries. Regarding the number of protein-coding genes, *C. kurzii* (82) had fewer than *C. velutina* and *C. decandra* (both 85) but more than *C. glomerata* (80). Notably, there were some differences in the types of tRNA genes among the four species (see Table S1). For example, *C. glomerata* lacked *trnG-UCC* and *trnI-GAU*, while its trnM-CAU gene was present in four copies, potentially reflecting variation in translational regulation or adaptive evolution.

The IR regions of *C. kurzii* also contained 17 duplicated genes (Table 3, see Table S2), including 4 rRNA genes, 6 protein-coding genes (*rpl23*, *rpl2*, *rps7*, *rps12*, *ndhB*, and *ycf2*), and 7 tRNA genes. Among all genes, 14 genes contained one intron, while 3 genes (*rps12*, *clpP*, and *ycf3*) contained two introns.

### 2.2. Dispersed Repeats and SSR Analysis

**Figure 3:**
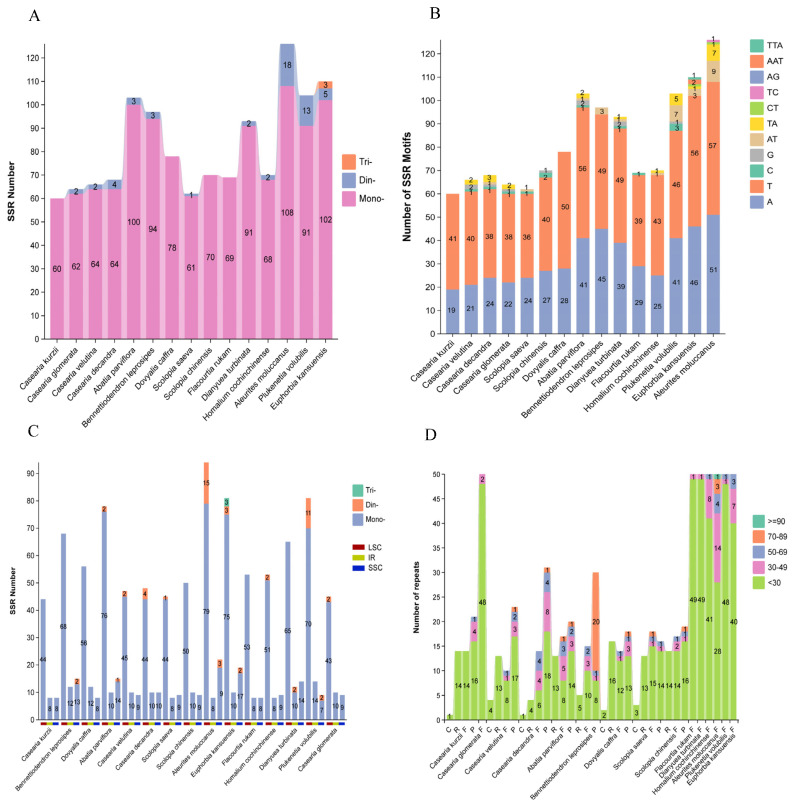
Identification of dispersed repeat sequences and SSRs in the chloroplast genomes of 15 species. The x-axis represents species names, and the y-axis represents the number of repetitive sequences or motifs. (**A**) The number of SSRs in the chloroplast genomes of these species. (**B**) The number of SSRs with different motifs. (**C**) The distribution of SSRs in the LSC, SSC, and IR regions. The red, yellow, and blue blocks represent the LSC, IR, and SSC regions, respectively. (**D**) The number of dispersed repeat sequences of different lengths in these chloroplast genomes. C: Complementary repeat, R: Reverse repeat, F: Forward repeat, P: Palindromic repeat.

Using REPuter, we analyzed the chloroplast genomes of *C. kurzii* and 14 closely related species to identify dispersed repeat sequences longer than 1 bp. A total of 750 dispersed repeats were detected and classified into four types: forward (10–50), palindromic (0–31), reverse (0–16), and complementary (0–4) repeats (Figure 3D). The majority of these repeats (81.2%) were shorter than 30 bp, followed by repeats ranging from 30 to 39 bp (10.0%), while repeats ≥90 bp were the least frequent (0.1%). Notably, *C. kurzii* exhibited significantly fewer complementary repeats compared to the other repeat types, suggesting unique structural or evolutionary features within its chloroplast genome.

Simple sequence repeat (SSR) analysis identified a total of 1240 SSRs across the 15 chloroplast genomes (Figure 3A). These SSRs consisted exclusively of mononucleotide (95.32%), dinucleotide (4.43%), and trinucleotide (0.25%) repeats. Among them, the total SSR counts ranged from 60 to 126, with *C. kurzii*, *D. caffra*, *S. chinensis*, and *F. rukam* containing only mononucleotide repeats.

Further examination of SSR motifs (Figure 3B) revealed that the 1182 mononucleotide repeats were predominantly A/T-based (98.14%). Among the 55 dinucleotide repeats, AT/TA motifs were the most common (92.73%), whereas CT, TC, and AG repeats occurred infrequently. The three trinucleotide repeats—two AAT and one TTA—were exclusively found in *E. kansuensis*.

Regional distribution analysis of SSRs (Figure 3C) indicated that most SSRs (74.36%) were located in the LSC region, followed by the SSC (13.35%) and IR regions (12.29%). In the *C. kurzii* chloroplast genome, 41 SSRs (74.55%) were found in intergenic spacers (IGSs), while 7 were located within introns of *ndhA*, *rpl16*, *petD*, *clpP*, *rpoC1*, and *atpF*. Another 7 were identified in the coding regions of *rpoC2*, *rpoC1*, *rpoB*, *cemA*, *ndhF*, and *ycf1*, with *ycf1* containing two SSRs (Table S3). This distribution pattern suggests a predominance of SSRs in non-coding regions, potentially implicating these repeats in regulatory functions.

### 2.3. Codon Usage Analysis

**Figure 4:**
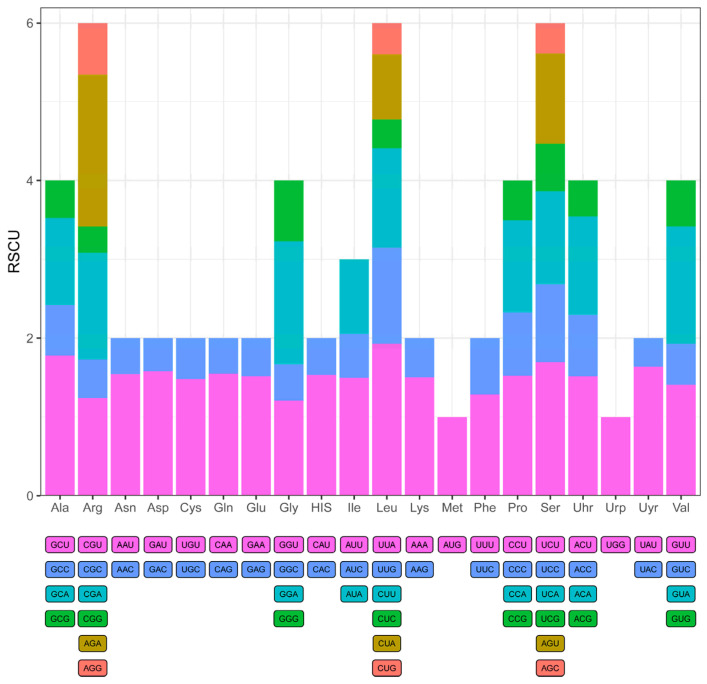
Relative synonymous codon usage (RSCU) values of all protein-coding genes in the chloroplast genome of *C. kurzii*. The x-axis represents amino acids, while the y-axis denotes RSCU values. Different colored blocks correspond to different codons.

To assess the potential impact of codon usage on gene expression and evolutionary adaptation, we analyzed the codon usage frequency and relative synonymous codon usage (RSCU) values for protein-coding genes in the *C. kurzii* chloroplast genome (Figure 4). Across all protein-coding sequences, 61 codons (excluding stop codons) were utilized, representing 20 amino acids. Among these, leucine (Leu, L), arginine (Arg, R), and serine (Ser, S) were encoded by six codons each, making them the most frequently represented amino acids. In contrast, methionine (Met, M) and tryptophan (Trp, W) were each encoded by a single codon (AUG and UGG, respectively) and exhibited relatively low usage frequencies.

Further analysis revealed that *C. kurzii* preferentially utilized UUA for leucine, AGA for arginine, and UCU for serine. Overall, most amino acids exhibited RSCU values greater than 1, indicating a significant codon usage bias. This codon preference may be associated with environmental adaptation, gene expression regulation, and long-term evolutionary selection pressures, providing a basis for further research on the adaptive evolution and functional regulation of genes in *C. kurzii*.

### 2.4. Sequence Divergence Analysis

**Figure 5:**
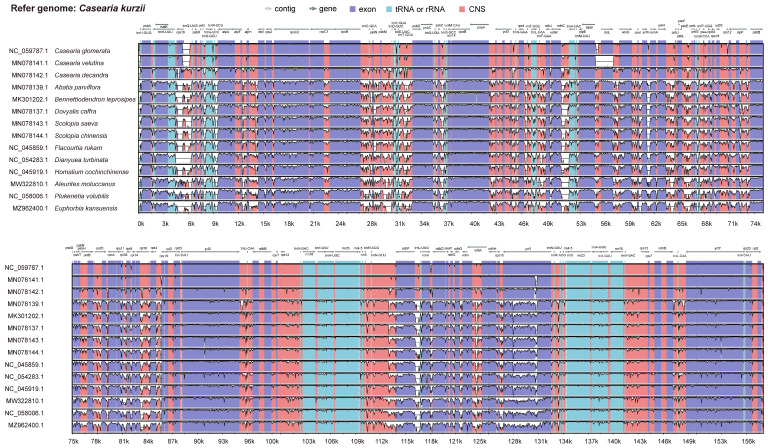
Percent identity plot comparing 15 chloroplast genomes using mVISTA. The x-axis represents the base sequence position, while the y-axis shows sequence identity ranging from 50% to 100%. Coding exons and untranslated regions (UTRs) are marked with different colored rectangles. Conserved regions are highlighted beneath the curves: red indicates conserved noncoding regions, blue represents conserved exons, and turquoise marks conserved UTRs. The direction of gene transcription is indicated by gray arrows. Pink bars represent noncoding sequences (CNS), while purple bars indicate exons.

We conducted a comparative analysis of the chloroplast genomes of 15 species using mVISTA, with the chloroplast genome of *C. kurzii* as the reference. The results revealed that while the overall genome structure was highly conserved, significant sequence divergence was observed in the large single-copy (LSC) region, whereas the small single-copy (SSC) and inverted repeat (IR) regions exhibited relatively fewer variations (Figure 5).

Within protein-coding regions, most genes were highly conserved across species, except for notable variations in *rps16*, *rbcL*, *clpP*, *petD*, *rpl16*, *ndhF*, *ndhA*, and *ycf1*. The highest sequence divergence was detected in intergenic spacer regions, particularly at the following loci: *rps16-trnQ-UUG*, *psbI-trnG-UCC*, *trnR-UCU-atpA*, *atpF-atpH*, *rpoB-trnC-GCA*, *psbM-trnD-GUC*, *trnT-GGU-psbD*, *psbZ-trnG-GCC*, *ndhC-trnV-UAG*, *ycf4-cemA*, *petA-psbJ*, *psbE-petL*, *and ndhF-trnL-UAG*. These highly variable intergenic regions may play a crucial role in phylogenetic differentiation and adaptive evolution, potentially contributing to genetic diversity and ecological adaptation among species.

### 2.5. Expansion and Contraction of IR Boundaries

**Figure 6:**
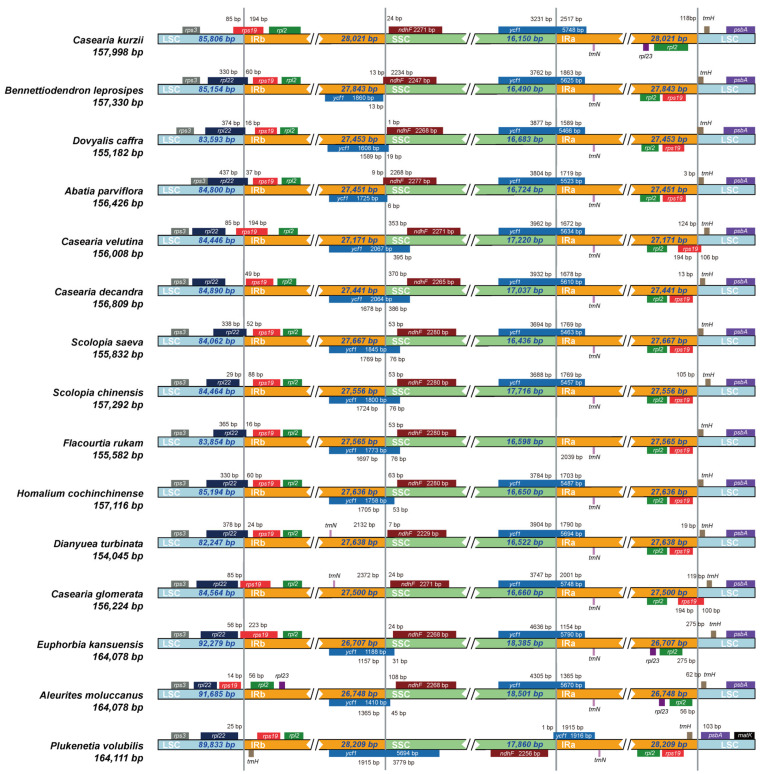
Comparison of LSC, IR, and SSC border regions among 15 chloroplast genomes. The light blue, yellow, and green blocks represent the LSC, IR, and SSC regions, respectively. Each row represents a species, with the species name and chloroplast genome length labeled on the left. The colored boxes above or below the main line indicate genes adjacent to these boundaries.

We further examined the expansion and contraction of IR/SC boundaries among the 15 species (Figure 6). The results indicated that changes in IR boundaries not only influence genome structure but can also impact gene expression and function [27]. In most species, the LSC-IRb boundary contained the *rpl22* and *rps19* genes. However, no homologous sequence or pseudogene remnant of the *rpl22* gene was detected in *C. kurzii*. Whole-genome sequence divergence analysis revealed that this gene was completely absent in the chloroplast genome of *C. kurzii*. Additionally, in *C. velutina*, *C. glomerata*, and *C. kurzii*, the *rps19* gene extended approximately 85 bp across the LSC-IRb boundary, whereas in *E. kansuensis*, *rps19* was positioned approximately 56 bp away from the boundary. Since *rps19* encodes a ribosomal small subunit protein, such positional shifts may influence translation efficiency or regulatory mechanisms.

In most species, the SSC-IRb boundary included the *ycf1* and *ndhF* genes. However, in *C. kurzii*, *D. turbinata*, and *C. glomerata*, no *ycf1* fragment was detected at this boundary, suggesting that the gene was primarily located in the IRa region or elsewhere. Conversely, in *B. leprosipes*, *ycf1* was predominantly positioned within the IRb region, located only 13 bp from the boundary. The *ndhF* gene was generally found within the SSC region, with distances from the boundary ranging from 1 bp to 370 bp. However, in *B. leprosipes* and *A. parviflora*, *ndhF* extended into the IRb region, whereas in *P. volubilis*, it was positioned at the SSC-IRa boundary. Notably, the orientation of *ycf1* in *P. volubilis* was opposite to that observed in most species, potentially due to an inverted repeat within the IR region.

The IRa-LSC boundary typically contained the *trnH* and *rps19* genes. However, in *C. kurzii*, *E. kansuensis*, and *A. moluccanus*, *rps19* was not detected at this boundary, suggesting that it may have undergone translocation or loss in these species. Structural variations in IR/SC boundaries may influence transcription and translation efficiency, as expansion or contraction of the IR region can lead to gene loss or transboundary distribution, thereby altering the structural and functional regulatory networks of the chloroplast genome. For instance, the absence of *rpl22* in *C. kurzii* may affect ribosomal protein assembly and photosynthetic regulation, whereas positional shifts of *ycf1* and *ndhF* in *P. volubilis* may be linked to ecological adaptation.

### 2.6. Nucleotide Polymorphism Analysis

**Figure 7:**
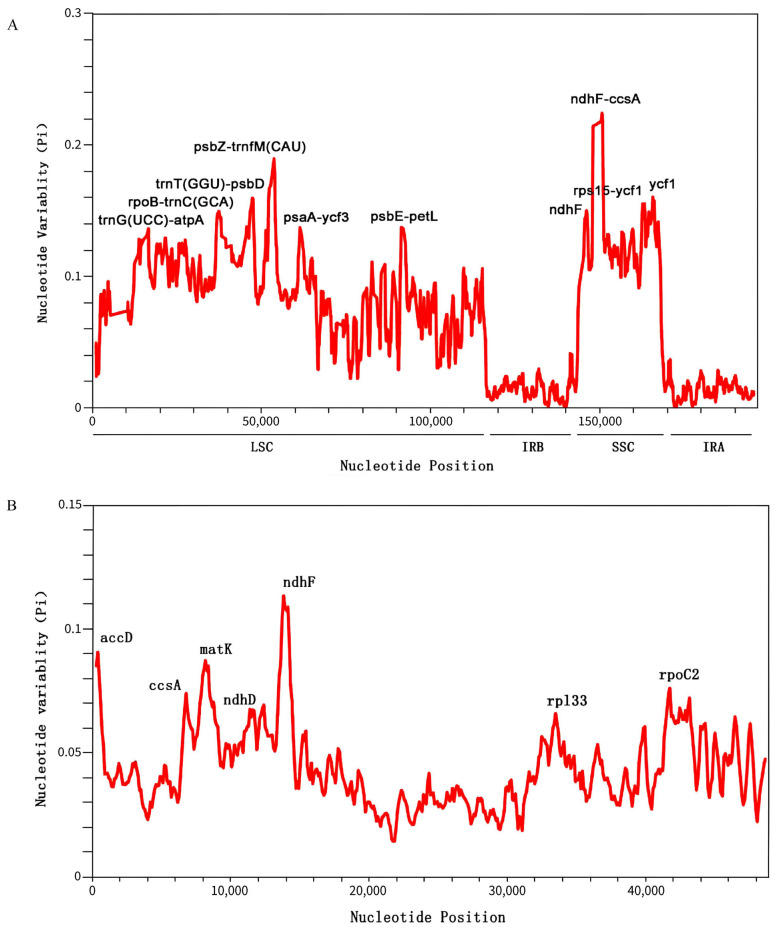
Nucleotide polymorphism (Pi) analysis of *C. kurzii* and 14 closely related species. (**A**) Sliding window analysis of nucleotide diversity (Pi) across the whole chloroplast genomes of 15 species. (**B**) Sliding window analysis of nucleotide diversity in 62 common protein-coding genes. The x-axis represents the midpoint position of each window, while the y-axis indicates the nucleotide diversity (Pi) of each window.

We assessed nucleotide polymorphism (Pi) in the chloroplast genomes of 15 species using a sliding window analysis of the entire genome and 62 single-copy coding sequences (CDSs). The genome-wide sliding window analysis identified multiple highly variable regions (Figure 7A), with Pi values ranging from 0 to 0.22 and an average of 0.06. The highest polymorphism was observed in the *ndhF-ccsA* region (Pi ≈ 0.22), followed by the *psbZ-trnfM(CAU)* region (Pi ≈ 0.19). These hypervariable variable sites were primarily located in the LSC and SSC regions, particularly in the following loci: *trnG(UCC)-atpA*, *rpoB-trnC(GCA)*, *trnT(GGU)-psbD*, *psbZ-trnfM(CAU)*, *psaA-ycf3*, and *psbE-petL* (LSC region), as well as *ndhF*, *ndhF-ccsA*, *rps15-ycf1*, and *ycf1* (SSC region). No significantly variable sites were detected in the IR regions.

We identified ten highly variable regions as potential molecular markers, including eight intergenic spacer regions and two protein-coding genes (*ndhF* and *ycf1*). These molecular markers are valuable for population genetics studies and conservation strategies for *C. kurzii*. Notably, coding region markers are particularly useful for assessing adaptive variation among populations, while non-coding region markers can aid in phylogeographic studies and genetic diversity analyses.

For the single-copy CDS analysis, we selected 62 single-copy CDSs from the 15 species and conducted multiple sequence alignments using MAFFT (Figure 7B). The nucleotide polymorphism (Pi) ranged from 0 to 0.11, with seven genes exhibiting relatively high variability: *accD*, *ccsA*, *matK*, *ndhD*, *ndhF*, *rpl33*, and *rpoC2*. Among these, *ndhF* displayed the highest polymorphism (Pi ≈ 0.11), followed by *accD* and *matK*. These highly variable genes serve as important molecular markers for further evolutionary analyses and species identification.

Based on these findings, we recommend developing species-specific primers targeting highly variable regions such as *ndhF-ccsA* and *psbZ-trnfM(CAU)* for the rapid identification of *Casearia* species. These molecular markers will facilitate ecological and conservation biology research on this genus and provide valuable support for phylogenetic studies.

### 2.7. Selection Pressure Analysis

To assess the impact of natural selection on gene evolution, we conducted a selection pressure analysis on 62 single-copy coding genes from 15 species. Using the branch-site model in PAML, we calculated the ratio of the nonsynonymous substitution rate (dN) to the synonymous substitution rate (dS) (ω = dN/dS). Typically, an ω value greater than 1 indicates positive selection, ω = 1 suggests neutral evolution, and ω < 1 signifies purifying or negative selection [28].

**Figure 8:**
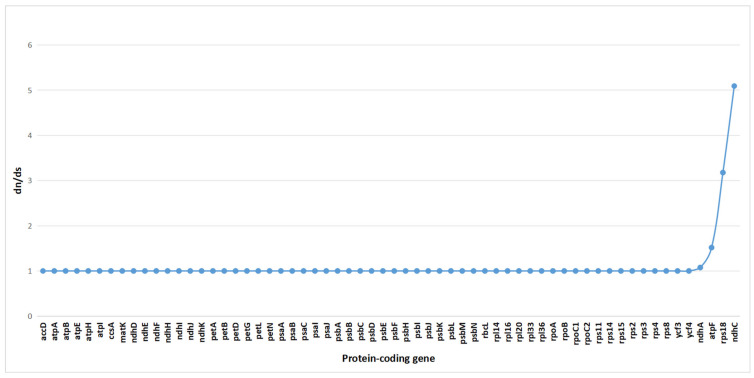
Selective pressure analysis of 62 common protein-coding genes in the chloroplast genomes of 15 species.

The results revealed that among the 62 single-copy genes in *C. kurzii*, 58 genes had ω values close to 1, indicating that they evolved primarily under neutral selection (Figure 8). This suggests that the proportion of nonsynonymous to synonymous substitutions was approximately equal, with no strong evidence of positive or negative selection. However, four genes (*ndhA*, *atpF*, *rps18*, and *ndhC*) exhibited ω values greater than 1, suggesting that they may have undergone strong selection pressure, potentially indicative of positive selection. These genes might play crucial roles in the adaptive evolution of *C. kurzii*.

Given the medicinal importance of *C. kurzii* in Yunnan, further investigation into the functions of these genes may provide insights into how the species responds to environmental pressures and maintains population dynamics at the molecular level. Exploring the roles of these genes in adaptive evolution is essential for understanding how *C. kurzii* copes with ecological challenges.

### 2.8. Phylogenetic Tree Reconstruction

**Figure 9:**
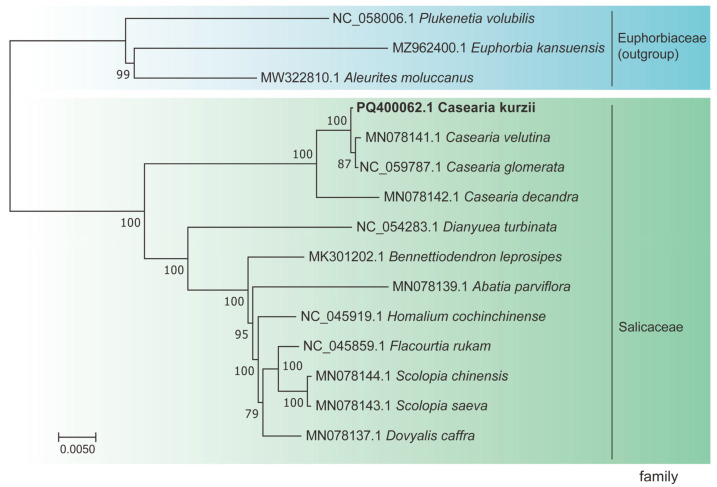
Phylogenetic relationships of 15 species inferred using maximum likelihood (ML) analysis. The ML tree was constructed based on 15 protein-coding genes (CDS). Species sequenced in this study are shown in bold.

To elucidate the phylogenetic relationships within the genus *Casearia*, we conducted a phylogenetic analysis using nucleotide sequences from 58 single-copy genes that exhibited neutral evolution. These genes were selected based on sequence coverage and quality to ensure the accuracy of phylogenetic inference. A maximum likelihood (ML) phylogenetic tree was constructed (Figure 9).

The results revealed that species within the same genus formed distinct clades, with strong support values for each evolutionary branch. Specifically, the three outgroup species formed independent branches, while the 12 species within Salicaceae clustered into a single major clade with a bootstrap support value of 100%, demonstrating robust evolutionary relationships. Following divergence from a common ancestor, *C. kurzii*, *C. decandra*, *C. velutina*, and *C. glomerata* formed a monophyletic group. Within this clade, *C. velutina* and *C. glomerata* were identified as sister taxa closely related to *C. kurzii*, with a bootstrap support value of 100%. Additionally, *C. decandra* clustered with these three *Casearia* species into a single branch, further supported by high confidence values, suggesting a close phylogenetic relationship among them.

### 2.9. Divergence Time Tree

**Figure 10:**
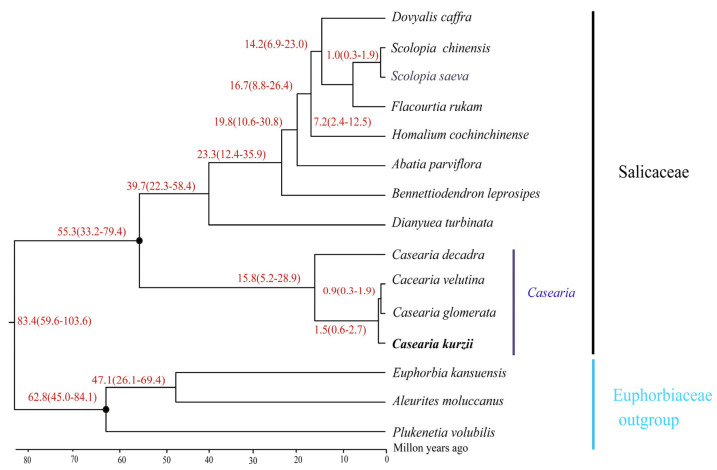
Estimated divergence time of *C. kurzii* based on nucleotide sequences of 58 shared single-copy protein-coding genes from 15 chloroplast genomes. Calibration groups are marked with black dots at corresponding nodes. The average divergence time for each node is shown in red, with numbers in parentheses indicating the 95% highest posterior density (HPD) interval, including minimum and maximum values. Species sequenced in this study are highlighted in bold.

Divergence time estimation indicated that the split between Salicaceae and Euphorbiaceae occurred approximately 83.4 million years ago (Mya) (95% highest posterior density [HPD]: 59.6–103.6 Mya) (Figure 10). The estimated crown ages for Salicaceae and Euphorbiaceae were 55.3 Mya (95% HPD: 33.2–79.4 Mya) and 62.8 Mya (95% HPD: 45.0–84.1 Mya). Within Salicaceae, two major clades diverged around 55.3 Mya (95% HPD: 33.2–79.4 Mya), while diversification within genera such as *Dianyuea* and *Bennettiodendron* occurred at approximately 39.7 Mya (95% HPD: 22.3–58.4 Mya).

For the genus *Casearia*, divergence among the four studied species (*C. kurzii*, *C. decandra*, *C. velutina*, and *C. glomerata*) was estimated at approximately 15.8 Mya (95% HPD: 5.2–28.9 Mya), marking the beginning of diversification within the group. Notably, *C. kurzii* diverged around 1.5 Mya (95% HPD: 0.6–2.7 Mya). These findings enhance our understanding of the evolutionary history of *Casearia* species and provide valuable insights into their biodiversity and ecological adaptation. These divergence time estimates not only establish a foundation for further studies on the evolutionary relationships within Salicaceae but also serve as crucial references for investigating their ecological adaptation mechanisms.

## 3. Materials and Methods

### 3.1. Plant Materials and DNA Extraction

In this study, three *C*. *kurzii* plants were sampled, and more than ten mature leaves were collected in total. Immediately after collection, the samples were frozen in liquid nitrogen and stored at −80 °C to preserve the integrity of the genomic DNA. All leaf samples were collected from Huashan Township, Jingdong County, Pu’er City, Yunnan Province, China (101°11′31″ E, 24°26′51″ N). The voucher specimens are stored at the Institute of Botany, Chinese Academy of Sciences. Total genomic DNA was extracted using the HiPure SF Plant DNA Mini Kit (Magen, Shanghai, China).

### 3.2. DNA Sequencing, Assembly, and Annotation

The quality and integrity of the extracted DNA were assessed using 1% agarose gel electrophoresis and UV spectrophotometry. A sequencing library was prepared using 300 ng of high-quality genomic DNA, which was fragmented to approximately 350 bp, purified, and sequenced on the Illumina NovaSeq 6000 platform (Illumina, San Diego, CA, USA) with a paired-end read length of 2 × 150 bp. All sequencing and related experimental procedures were conducted by Shanghai Yuanxin Biomedical Technology Co., Ltd. (Shanghai, China).

For quality control, FastQC v0.11.4 [29] was used to evaluate the raw sequencing data. Low-quality reads, adapter sequences, and reads containing excessive ambiguous bases (“N”) were removed using Cutadapt [30]. The resulting high-quality reads were used for subsequent assembly and downstream analyses.

The complete chloroplast genome sequence was obtained through de novo assembly using GetOrganelle v1.7.5+ [31], with *C. velutina* (GenBank accession: MN078141.1) as the reference. Assembly results were visualized and refined using Bandage v0.9.0 [32]. Chloroplast genome annotation was conducted using the GeSeq online platform [33] and manually curated with Geneious Prime 2021.1.1. The finalized chloroplast genome sequence was deposited in the NCBI database under accession number PQ400062.1. A schematic representation of the chloroplast genome was generated using OGDRAW v1.3.1 [34]. The raw sequencing data were also submitted to the NCBI Sequence Read Archive (SRA) under accession number SRR30619610.

### 3.3. Repeat Sequence Analysis

Simple sequence repeats (SSRs) were identified using MISA v2.1 [35]. Detection parameters were set as follows: a minimum of 10 repeat units for mononucleotides, 6 for dinucleotides, and 5 for tri-, tetra-, penta-, and hexanucleotides. The maximum allowed distance between two SSRs was set to 100 bp. For dispersed repeat analysis, REPuter [36] was used to identify repeat size and location, with the minimum repeat length set to 8 bp and a Hamming distance of 3. The detected repeat sequences were classified into four types: forward, reverse, complementary, and palindromic repeats.

### 3.4. Codon Usage Bias Analysis

Protein-coding gene sequences of the chloroplast genome were extracted using PhyloSuite v1.2.2 [37]. Codon usage frequency was analyzed with CodonW v1.4.2 [38], and relative synonymous codon usage (RSCU) values were calculated. A custom R script was used to visualize the RSCU results and assess codon usage preferences. Generally, an RSCU value greater than 1 indicates a preferred codon, a value of 1 suggests random usage, and a value less than 1 denotes underrepresented codon usage [39].

### 3.5. Comparative Genomic Analysis

Based on the phylogenetic classification of Salicaceae and their subtropical distribution, three *Casearia* species and eight other Salicaceae species were selected for comparative chloroplast genome analysis. Additionally, since Salicaceae is the sister group to Euphorbiaceae [40,41], three Euphorbiaceae species were included as outgroups. Complete chloroplast genome sequences of 14 species were retrieved from NCBI (Table S4). Structural variations at the SC/IR boundary regions were analyzed using IRscope [42]. Sequence divergence among multiple species was assessed using mVISTA [43] under the Shuffle-LAGAN model, with the annotated *C. kurzii* chloroplast genome as the reference. Multiple sequence alignments were performed using MAFFT v7.310 [44]. Nucleotide polymorphism analysis was conducted using DnaSP v6.12 [45] with a sliding window size of 600 bp and a step size of 100 bp.

### 3.6. Phylogenetic Analysis

A total of 11 species from Salicaceae and three from Euphorbiaceae were retrieved from NCBI for phylogenetic analysis. Sixty-two shared single-copy coding sequences (CDSs) were selected. First, selection pressure on these genes was assessed using PAML v4.9j [46]. The sequences were aligned with MAFFT v7.310, and poorly aligned regions were removed using Gblocks v0.91b. A maximum likelihood (ML) phylogenetic tree was constructed using RAxML v8.2.12 [47] with 500 bootstrap replicates under the GTRCATI nucleotide substitution model to ensure tree reliability.

### 3.7. Divergence Time Estimation

Divergence time estimation for *C. kurzii* was performed using the MCMCTree module in PAML v4.9j, based on 58 shared single-copy CDSs from 15 chloroplast genomes. Due to the lack of direct fossil evidence, calibration points for Salicaceae and Euphorbiaceae were obtained from literature searches in the TimeTree database [48]. The Markov chain Monte Carlo (MCMC) analysis was run for 80,000 generations, sampling every 10 generations, with the first 25% of samples discarded to ensure result stability. The divergence time estimation was conducted under clock = 2 and model = 0, and the 95% highest posterior density (HPD) interval was calculated. The output file (out.txt) was analyzed using Tracer v1.7.2 [49] to evaluate parameter convergence, ensuring all effective sample size (ESS) values exceeded 200. After multiple runs, the most stable result was selected, and a maximum clade credibility tree with median divergence times and 95% HPD intervals was generated using TreeAnnotator v2.1.2. The final phylogenetic tree was visualized and edited in FigTree v1.4.4 [50].

## 4. Discussion

### 4.1. Chloroplast Genome Structure and Basic Characteristics

The chloroplast genome is generally highly conserved, exhibiting a relatively low evolutionary rate, making it a valuable resource for plant molecular systematics and ecological studies [51,52]. This stability is particularly valuable for elucidating the genetic relationships and evolutionary characteristics of medicinal plants, providing more precise phylogenetic insights [53]. In this study, we sequenced and assembled the complete chloroplast genome of *C. kurzii* using next-generation sequencing (NGS) technology. Comparative analyses with other *Casearia* species, including *C. glomerata* and *C. velutina*, revealed significant structural and sequence variations.

Our comparative analysis showed that *C. kurzii* retained the typical quadripartite structure, consistent with other *Casearia* species (*C. glomerata* and *C. velutina*) available in the NCBI database. The GC content and gene composition of *C. kurzii* were highly conserved. The total length of the *C. kurzii* chloroplast genome (157,998 bp) was slightly larger than that of *C. glomerata* (156,806 bp) and *C. velutina* (156,008 bp) [25,26], indicating subtle structural variations within the genus.

### 4.2. Codon Usage and Selection Pressure

Codon usage bias not only reflects species adaptation to environmental changes and evolutionary processes but is also closely associated with gene expression regulation [54,55]. Analysis of protein-coding genes in the *C. kurzii* chloroplast genome revealed that among the 61 sense codons (excluding stop codons), 20 amino acids were encoded. Leucine (Leu, L), arginine (Arg, R), and serine (Ser, S) were the most frequently represented amino acids, each encoded by six codons. In contrast, methionine (Met, M) and tryptophan (Trp, W) were each encoded by a single codon (AUG and UGG, respectively), exhibiting relatively low usage frequencies.

The relative synonymous codon usage (RSCU) values indicated that *C. kurzii* preferentially utilized UUA for leucine, AGA for arginine, and UCU for serine. Most amino acid codons had RSCU values greater than 1, demonstrating significant codon usage bias. This pattern is consistent with observations in other plant species, such as *Hypericum addingtonii*, where leucine and serine are also preferentially used [56], suggesting a conserved codon selection mechanism across species. Overall, codon usage preferences in *C. kurzii* are likely shaped by a combination of mutational bias, natural selection, and long-term evolutionary pressures, further supporting the close relationship between codon usage, gene function, and environmental adaptation.

To access selection pressure, we analyzed 62 single-copy protein-coding genes across 15 species, including *C. kurzii*. Using the branch-site model in the PAML software, we calculated the ratio of nonsynonymous (dN) to synonymous (dS) substitution rates (ω = dN/dS). The results showed that most genes (58 out of 62) had ω values close to 1, indicating neutral evolution or a relatively balanced selection state [57]. However, four genes—*ndhA*, *atpF*, *rps18*, and *ndhC*—exhibited ω values greater than 1, indicating positive selection, and a potential role in the adaptive evolution of *C. kurzii*.

Notably, *ndhA* and *ndhC* are closely linked to electron transport and photosynthesis, while *atpF* encodes a subunit involved in ATP synthase complex assembly and function, which is critical for energy efficiency and stress responses in plants. Similarly, *rps18*, which participates in ribosome assembly and protein synthesis, may confer an adaptive advantage under environmental stress. Collectively, these genes may have played a crucial role in enabling *C. kurzii* to thrive in the diverse and complex ecological conditions of Yunnan. Further functional studies on these genes could provide deeper insights into their molecular mechanisms in environmental stress responses and population dynamics.

### 4.3. Repetitive Sequences and Identification of Highly Variable Regions

Notably, *C. kurzii* exhibited an exceptionally high abundance of mononucleotide simple sequence repeats (SSRs), with 60 identified, all composed of A/T motifs. Among these, 74.55% were located in intergenic spacer (IGS) regions, showing a distinct distribution pattern compared to other species in the same family [58]. Given their high polymorphism and susceptibility to mutations [59,60], SSRs are widely recognized as effective molecular markers for population genetics and species identification, as observed in *C. grandiflora* [23]. Our study further confirmed that the majority of SSRs in *C. kurzii* were concentrated in non-coding regions, suggesting a potential role in transcriptional regulation and genomic stability.

In addition to SSRs, various types of dispersed repeat sequences were identified in the chloroplast genome, which play a crucial role in maintaining genome structure and stability [61,62]. Our analysis revealed that *C. kurzii* shared similar dispersed repeat patterns with *C. glomerata*, yet displayed notable divergence from other closely related species. This interspecific variation offers valuable insights for comparative chloroplast genomic research and supports the phylogenetic resolution within the genus *Casearia*.

The comparative analysis of nucleotide polymorphism and sequence divergence in *C. kurzii* and closely related species in this study revealed eight highly variable regions and eight genes with high nucleotide diversity, which serve as molecular markers for future phylogenetic analysis and species identification within *Casearia*. Protein-coding regions such as *ndhF*, *ycf1*, and *matK* have been proposed as key reference markers for species identification [63]. Additionally, regions like *ndhF-ccsA*, *psbZ-trnfM*, *ndhF*, *accD*, and *matK* exhibited the highest nucleotide polymorphism in our study, highlighting their potential as candidate markers for molecular identification and evolutionary investigations in *C. kurzii* [64,65]. For instance, the *ycf1* gene region exhibited significant variation in *C. kurzii*, potentially linked to plastid ATP synthase function [66]. Additionally, the expansion and contraction of inverted repeat (IR) boundaries may contribute to variations in gene copy numbers, a key feature of chloroplast genome evolution that reflects dynamic genome rearrangement [67,68]. Compared to previous sequencing of *C. glomerata* [25], our study uncovered a unique phenomenon in *C. kurzii*, involving the deletion and/or translocation of *rps19* and *rpl22* at the LSC/IRb boundary. The *rpl22* gene encodes the chloroplast ribosomal large subunit protein L22, which is crucial for maintaining normal chloroplast translation function [69]. Although its loss in some angiosperms can be compensated by nuclear genes [70], it is unknown whether such a mechanism exists in the *C. kurzii* nuclear genome. The absence of *rpl22* may affect physiological processes such as photosynthesis in *C. kurzii*, and these findings could be related to the species’ geographical distribution and ecological adaptation mechanisms. Furthermore, *ycf1* gene is widely involved in essential functions such as chloroplast protein transport [71,72], and the loss of its boundary suggests that *C. kurzii* may have evolved a novel molecular mechanism for chloroplast protein transport, distinct from the classical pathway.

Species within the genus *Casearia* are often morphologically similar and display fragmented geographic distributions [21,22]. The highly variable SSRs and markers such as *ndhF* identified in this study provide valuable tools for population genetics and species identification, especially for under-studied taxa in regions such as Madagascar, the Brazilian Cerrado, and parts of Asia [73,74]. These markers enable the differentiation of morphologically similar species, facilitate the monitoring of population dynamics, and support the implementation of targeted conservation strategies. Additionally, their association with traits such as growth rate and stress tolerance makes them useful for germplasm screening and breeding. Future population genetic studies leveraging these markers could provide key insights into local adaptation and responses to environmental change, thereby contributing to both evolutionary research and the conservation management of *Casearia* species.

### 4.4. Phylogenetic and Evolutionary Analysis

From a phylogenetic perspective, our analysis revealed that *C. kurzii*, *C. decandra*, *C. velutina*, and *C. glomerata* formed a strongly supported monophyletic clade (100% bootstrap support), consistent with previous studies that identified a closely related cluster of *C. velutina*, *C. glomerata*, and *C. decandra* [25,26]. By incorporating *C. kurzii* into this lineage, we estimated its divergence time to be approximately 15.8 million years ago (Mya) using molecular clock analysis. While previous estimates of *Casearia* divergence times vary (e.g., 12.09 Mya, 40 Mya), our results generally support the hypothesis that multiple evolutionary branching events occurred from the Miocene to the Pliocene [26].

However, some studies have reported discrepancies in divergence time estimates. For instance, Ogutcen et al. [75] proposed that *Casearia* began diversifying around 40 Mya based on different datasets and fossil calibrations. Additionally, de Mestier et al. [24] estimated the crown age of *Casearia* at approximately 39 Mya, with *C. decandra* diverging around 12.09 Mya, closely aligning with our estimate of 15.8 Mya. However, their divergence estimates for *C. velutina* (5.77 Mya vs. 0.9 Mya) showed substantial variation. These inconsistencies may stem from differences in molecular data selection, taxon sampling, and calibration settings [76]. To resolve these discrepancies, future studies should integrate whole-genome data and ecological evidence to refine estimates of *Casearia* origins and global distribution patterns.

## 5. Conclusions

In this study, we assembled and annotated the complete chloroplast genome of *C*. *kurzii* and performed comparative analyses with closely related species within the genus. The results revealed a high degree of structural conservation across *Casearia* chloroplast genomes. Distinctive genomic features of *C. kurzii*, including the loss of the *rpl22* gene and the presence of unique single-nucleotide SSRs, were identified. Five candidate molecular markers were proposed for accurate species-level identification. Phylogenetic analysis and divergence time estimation positioned *C. kurzii* as diverging from its closest relatives approximately 15.8 million years ago. These findings provide valuable genomic resources for species identification, phylogenetic classification, and further evolutionary studies of *Casearia*.

## Figures and Tables

**Table 1:** Major taxonomic studies and viewpoints on the genus *Casearia*.

Author (Year)	Taxonomic Treatment or Viewpoint on *Casearia*	Representative Conclusions or Changes	Reference
Tulasne (1868)	First conducted a systematic study of Malagasy *Casearia*, describing *C. lucida*, *C. nigrescens*, *C. amplissima*, *C. parvifolia*, *C. elliptica*, etc.	Established several new species based on morphological traits (e.g., leaf size and shape), laying the foundation for subsequent taxonomic research on this genus in Madagascar.	Tulasne, 1868 [14]
Perrier de la Bâthie (1940, 1946)	In his treatment of the Flore de Madagascar, continued to subdivide Malagasy *Casearia*, mainly adopting and revising Tulasne’s classification; added several new varieties (var. onivensis, var. ovata, var. subtrinervia, etc.) and new combinations based on *C. nigrescens* and others.	Continued to treat multiple “small-leaf” taxa as varieties under *C. nigrescens*, and revised species such as *C. amplissima* and *C. parvifolia* (= *C. tulasneana*). Provided substantial morphological evidence for classifying this genus in Madagascar at that time.	Perrier de la Bâthie, 1940, 1946 [15,16]
Sleumer (1971, 1980)	In revising *Casearia* from the Africa–Madagascar–Mascarene region, tended toward extensive lumping: most previously described Malagasy species (including those named by Perrier) were merged into *C. nigrescens* (further divided into var. nigrescens and var. lucida).	Emphasized the high degree of floral similarity among Malagasy species, thereby merging most morphologically distinct species into *C. nigrescens*, resulting in extreme nomenclatural “lumping.” However, many specimens did not fully match Sleumer’s classification morphologically, prompting future reexamination.	Sleumer, 1971, 1980 [17,18]
Alford (2005)	Based on molecular phylogenetics, proposed separating Samydaceae (or including it in a broadly circumscribed Salicaceae) from the “old,” polyphyletic Flacourtiaceae. Within this framework, *Casearia* shares lineage features with related genera (*Laetia*, *Zuelania*, *Samyda*, etc.).	Molecular data support a close relationship between *Casearia* and several smaller genera (*Laetia*, *Samyda*, *Zuelania*, etc.), but sampling of most Malagasy species was not detailed at the time. Also noted that some species groups and boundary issues remain unresolved in this genus.	Alford, 2005 [19]
Samarakoon and Alford (2019)	Integrated molecular and morphological evidence to merge *Samyda*, *Laetia*, *Zuelania*, etc. into *Casearia*; clarified that *Casearia* spans multiple lineages between the Old World and New World, although sampling of Malagasy and African species remains limited.	Molecular phylogenetic analyses revealed that *Samyda*, *Laetia*, *Zuelania*, etc., previously separated in traditional classifications, are nested within *Casearia*, thus supporting a broader “*Casearia sensu lato*.” Nonetheless, further evolutionary and taxonomic reexaminations are needed for various African–Malagasy species.	Samarakoon and Alford, 2019 [20]
Applequist and Gates (2020)	Emphasized that Malagasy *Casearia* urgently requires further revision; described two new Malagasy species (*C. anosyensis* and *C. montigena*). Also reaffirmed that Sleumer’s broad lumping of numerous small-leaf taxa into *C. nigrescens* does not adequately reflect actual diversity.	Stressed a comprehensive approach integrating morphology and taxonomy, confirming at least two new “small-leaf” species distinct from known taxa (e.g., *C. tulasneana*). Called for further molecular and micromorphological studies to refine classification of Malagasy and African *Casearia*; also conducted IUCN assessments (e.g., *C. anosyensis* was deemed Endangered).	Applequist and Gates, 2020 [22]

**Table 2:** Basic features of chloroplast genome of *Casearia*.

Species Names	*C. kurzii*	*C. velutina*	*C. decandra*	*C. glomerata*
Total length (bp)	157,998	156,008	156,809	156,224
LSC length (bp)	85,806	84,446	84,890	84,564
IRB length (bp)	28,021	27,171	27,441	27,500
SSC length (bp)	16,150	17,220	17,037	16,660
IRA length (bp)	28,021	27,171	27,441	27,500
Coding length (bp)	78,334	79,662	80,322	77,730
Non-coding length (bp)	79,664	76,346	76,487	78,494
Total number of genes	127	130	130	124
Protein-coding genes (duplicated)	82 (6)	85 (9)	85 (9)	80 (6)
tRNA genes (duplicated)	37 (7)	37 (7)	37 (7)	34 (7)
rRNA genes (duplicated)	8 (4)	8 (4)	8 (4)	8 (4)
Pseudo genes	0	1	1	0
GC content of genome (%)	36.72	36.83	36.8	36.81
GC content of LSC (%)	34.46	34.61	34.57	34.59
GC content of IRB (%)	42.05	42.38	42.27	42.19
GC content of SSC (%)	30.21	30.19	30.29	30.33
GC content of IRA (%)	42.05	42.38	42.27	42.19

**Table 3:** Genes annotated in the chloroplast genome of *C. kurzii*.

Gene Function	Gene Category	Name of Genes	Total Number
Self-replication	Large subunit of ribosome	*rpl20*, *rpl23(X2)*, *rpl14*, *rpl33*, rpl16 *, *rpl36*, rpl2 * (X2)	9
	Small subunit of ribosome	*rps11*, *rps14*, *rps15*, *rps16*, *rps2*, *rps3*, *rps18*, *rps4*, *rps19*, *rps7(X2)*, *rps8*, rps12 ** (X2)	14
	DNA dependent RNA polymerase	*rpoA*, *rpoB*, *rpoC1* *, *rpoC2*	4
	rRNA gene	*rrn5(X2)*, *rrn4.5(X2)*, *rrn16(X2)*, *rrn23(X2)*	8
	tRNA gene	*trnR-UCU*, *trnE-UUC*, *trnT-GGU*, *trnS-GGA*, *trnI-CAU(X2)*, *trnV-GAC(X2)*, *trnR-ACG(X2)*, trnL-UAA *, *trnG-GCC, trnD-GUC*, *trnY-GUA*, *trnP-UGG*, *trnM-CAU*, *trnL-CAA(X2)*, *trnS-GCU*, *trnW-CCA*, *trnF-GAA*, *trnT-UGU*, *trnS-UGA*, trnV-UAC *, trnG-UCC *, *trnL-UAG*, trnI-GAU * (X2), *trnH-GUG*, *trnfM-CAU*, *trnQ-UUG*, *trnN-GUU(X2)*, trnK-UUU *, trnA-UGC * (X2), *trnC-GCA*	37
Gene for photosynthesis	Subunits of photosystem I	*psaA*, *psaB*, *psaC, psaI*, *psaJ*	5
	Subunits of photosystem II	*psbL*, *psbZ*, *psbM*, *psbN*, *psbA*, *psbB*, *psbC*, *psbD*, *psbE*, *psbF*, *psbT*, *psbH*, *psbI*, *psbJ*, *psbK*	15
	Subunits of NADH-dehydrogenase	*ndhG*, *ndhH*, *ndhI*, *ndhJ*, *ndhK*, ndhA *, ndhB * (X2), *ndhC, ndhD*, *ndhE*, *ndhF*	12
	Subunits of cytochrome b/f complex	*petL*, *petN*, petA, petB *, petD *, *petG*	6
	Subunit for ATP synthase	*atpI*, *atpA*, *atpB, atpE*, atpF *, *atpH*	6
	Large subunit of rubisco	*rbcL*	1
Other genes	Maturase	*matK*	1
	Protease	clpP **	1
	Envelope membrane protein	*cemA*	1
	Subunit of Acetyl-carboxylase	*accD*	1
	C-type cytochrome synthesis gene	*ccsA*	1
Unknown function	Open reading frames (ORF,ycf)	*ycf1*, *ycf2(X2)*, ycf3 **, *ycf4*	5

Note: * Gene with one intron; ** Gene with two introns; (×2) Gene with two copies.

## Data Availability

The chloroplast genome sequence supporting this study has been uploaded to GenBank (National Center for Biotechnology Information) with the accession number PQ400062 and the SRA number is SRR30619610, respectively. All other data supporting this study, including figures’ raw data, analysis data, and phylogenetic tree files are openly available in Mendeley Data at https://doi.org/10.17632/8gxv9vhj25.1 (accessed on 10 January 2025).

## Supplementary Materials

The supporting information can be downloaded at: https://www.mdpi.com/article/10.3390/plants14091356/s1, Table S1: Genes annotated in the chloroplast genome of four *Casearia*; Table S2: Genes with introns in the *C. kurzii*; Table S3: The distribution of SSRs in the *C. kurzii* cp genome in protein coding, intronic and intergenic regions; Table S4: Genbank accession information for 14 species.

## Abbreviations

The following abbreviations are used in this manuscript:

LSC	large single-copy region
SSC	small single-copy region
IRs	inverted repeat regions
GC	guanine-cytosine
SSRs	simple sequence repeats
RSCU	relative synonymous codon usage
CNSs	conserved noncoding sequences
Pi	nucleotide diversity
ML	maximum likelihood
HPD	highest posterior density

## References

[1] Jensen P.E., Leister D. (2014). Chloroplast Evolution, Structure and Functions. F1000Prime Rep..

[2] Wicke S., Schneeweiss G.M., dePamphilis C.W., Müller K.F., Quandt D. (2011). The Evolution of the Plastid Chromosome in Land Plants: Gene Content, Gene Order, Gene Function. Plant Mol. Biol..

[3] Carvalho L.R., Nunes R., Sobreiro M.B., Dias R.O., Corvalán L.C.J., Braga-Ferreira R.S., Targueta C.P., Telles M.P.C. (2023). The Complete Chloroplast Genome Sequence of *Eugenia Klotzschiana* O. Berg Unveils the Evolutionary Dynamics in Plastomes of Myrteae DC. Tribe (Myrtaceae). Gene.

[4] Daniell H., Lin C., Yu M., Chang W. (2016). Chloroplast Genomes: Diversity, Evolution, and Applications in Genetic Engineering. Genome Biol..

[5] Liu Y., Liu K., Dong W., Dong S., Wang Y., Xu C., Li E., Sun J. (2025). Chloroplast Genome Evolution of Hamamelidaceae at Subfamily Level. Ecol. Evol..

[6] Jiang Y., Li H., Wu M., Zhang X., Baasanmukh S., Li H., Sun H., Chen S. (2025). Comparative Chloroplast Genomes of *Incarvillea* Species (Bignoniaceae) Unveiled Genomic Diversity and Shed Light on Phylogenetic Relationships. BMC Plant Biol..

[7] Nguyen P.A.T., Nguyen H.A., Do H.D.K., Tan Khang D. (2025). The Complete Chloroplast Genome of *Baccaurea Ramiflora Lour.* Cultivar Ha Chau (Phyllanthaceae, Malpighiales). Ecol. Evol..

[8] Ma J., Yang X., Zhang Q., Zhang X., Xie C., Tuerhong M., Zhang J., Jin D., Lee D., Xu J. (2019). Cytotoxic Clerodane Diterpenoids from the Leaves of *Casearia Kurzii*. Bioorg. Chem..

[9] iPlant Flora of China. http://www.iplant.cn/foc.

[10] An L., Ma J., Yang X., Liang Y., Wang H., Tuerhong M., Lall N., Abudukeremu M., Zhang Y., Lee D. (2020). Caseahomopene A, a Ring-Expanded Homotriterpenoid from *Casearia Kurzii* Showing Anti-Inflammatory Activities in Vitro and in Vivo. Bioorg. Chem..

[11] Liang Y., Zhang Q., Yang X., Li Y., Zhang X., Li Y., Du Q., Jin D., Cui J., Lall N. (2020). Diterpenoids from the Leaves of *Casearia Kurzii* Showing Cytotoxic Activities. Bioorg. Chem..

[12] Zhang L., Wang X., Wang T., Zhang J., Huang Z., Shen T., Lou H., Ren D., Wang X. (2020). Dolabellane and Clerodane Diterpenoids from the Twigs and Leaves of *Casearia Kurzii*. J. Nat. Prod..

[13] Shuo Y., Zhang C., Yang X., Liu F., Zhang Q., Li A., Ma J., Lee D., Ohizumi Y., Guo Y. (2019). Clerodane Diterpenoids from *Casearia Kurzii* and Their Cytotoxic Activities. J. Nat. Med..

[14] Tulasne L. (1868). Florae Madagascariensis fragmenta. Fragmentum tertium. Violarieae, Sauvagesieae, Turneraceae, Samydeae, et Bixaceae. Ann. Sci. Nat. Sér.

[15] Perrier de la Bâthie H. (1940). Révision des Flacourtiacées de Madagascar et des Comores. Mém. Mus. Natl. Hist. Nat..

[16] Perrier de la Bâthie H. (1946). Flacourtiacées. Flore de Madagascar et des Comores.

[17] Sleumer H. (1971). Le genre Casearia Jacq. (Flacourtiaceae) en Afrique, à Madagascar et aux Mascareignes. Bull. Jard. Bot. Nat. Belg..

[18] Sleumer H. (1980). Flacourtiaceae. Flora Neotropica.

[19] Alford M.H. (2005). Systematic Studies in Flacourtiaceae. Ph.D. Thesis.

[20] Samarakoon T., Alford M.H. (2019). New Names and Combinations in Neotropical Samydaceae. Novon.

[21] de Mestier A., Lücking R., Gutierrez J., Brokamp G., Celis M., Borsch T. (2023). Nested Singletons in Molecular Trees: Utility of Adding Morphological and Geographical Data from Digitized Herbarium Specimens to Test Taxon Concepts at Species Level in the Case of *Casearia* (Salicaceae). Ecol. Evol..

[22] Applequist W.L., Gates M.T. (2020). Two New Small-Leaved Species of *Casearia* (Salicaceae) from Madagascar. Novon.

[23] Costa M.F., Pereira A.A., Pinheiro J.B., Zucchi M.I., Araújo A.S.F., Gomes R.L.F., Valente S.E.S., Oliveira M.E.A., Lopes A.C.A. (2017). Chloroplast Diversity of *Casearia Grandiflora* in the Cerrado of Piauí State. Genet. Mol. Res..

[24] de Mestier A., Brokamp G., Celis M., Falcón-Hidalgo B., Gutiérrez J., Borsch T. (2022). Character Evolution and Biogeography of *Casearia* (Salicaceae): Evidence for the South American Origin of a Pantropical Genus and for Multiple Migrations to the Caribbean Islands. TAXON.

[25] Huang H., Xie Y., Luo S., Zhang L., Deng C., Chen Z. (2021). The Complete Chloroplast Genome Sequence of *Casearia Glomerata Roxb.* (Malpighiales; Salicaceae) from Fujian, China. Mitochondrial DNA B Resour..

[26] Li M.-M., Wang D.-Y., Zhang L., Kang M., Lu Z., Zhu R., Mao X., Xi Z., Ma T. (2019). Intergeneric Relationships within the Family Salicaceae *s.l.* Based on Plastid Phylogenomics. Int. J. Mol. Sci..

[27] Ma H., Liu Z., Lan W., Yang M., Mo Q., Huang X., Wu P., Huang H., Huang M. (2025). Complete Chloroplast Genomes of 9 *Impatiens* Species: Genome Structure, Comparative Analysis, and Phylogenetic Relationships. Int. J. Mol. Sci..

[28] Meng J., Wang Y., Song H., Dong W., Dong N. (2025). Insights into Phylogeny, Taxonomy, Origins and Evolution of *Crataegus* and *Mespilus*, Based on Comparative Chloroplast Genome Analysis. Genes.

[29] Andrews S. FastQC: A Quality Control Tool for High Throughput Sequence Data. https://www.bioinformatics.babraham.ac.uk/projects/fastqc/.

[30] Martin M. (2011). CUTADAPT Removes Adapter Sequences from High-Throughput Sequencing Reads. EMBnet.J..

[31] Jin J., Yu W., Yang J., Song Y., dePamphilis C., Yi T., Li D. (2020). GetOrganelle: A Fast and Versatile Toolkit for Accurate de Novo Assembly of Organelle Genomes. Genome Biol..

[32] Wick R.R., Schultz M.B., Zobel J., Holt K.E. (2015). Bandage: Interactive Visualization of de Novo Genome Assemblies. Bioinformatics.

[33] Tillich M., Lehwark P., Pellizzer T., Ulbricht-Jones E.S., Fischer A., Bock R., Greiner S. (2017). GeSeq—Versatile and Accurate Annotation of Organelle Genomes. Nucleic Acids Res..

[34] Greiner S., Lehwark P., Bock R. (2019). OrganellarGenomeDRAW (OGDRAW) Version 1.3.1: Expanded Toolkit for the Graphical Visualization of Organellar Genomes. Nucleic Acids Res..

[35] Beier S., Thiel T., Münch T., Scholz U., Mascher M. (2017). MISA-Web: A Web Server for Microsatellite Prediction. Bioinformatics.

[36] Kurtz S., Choudhuri J.V., Ohlebusch E., Schleiermacher C., Stoye J., Giegerich R. (2001). REPuter: The Manifold Applications of Repeat Analysis on a Genomic Scale. Nucleic Acids Res..

[37] Zhang D., Gao F., Jakovlić I., Zou H., Zhang J., Li W.X., Wang G.T. (2020). PhyloSuite: An Integrated and Scalable Desktop Platform for Streamlined Molecular Sequence Data Management and Evolutionary Phylogenetics Studies. Mol. Ecol. Resour..

[38] Niu T., Tian C., Yang Y., Liu Q., Liu L., Tao Q., Li Z., Wu Z. (2023). Complete Chloroplast Genome of *Corethrodendron Fruticosum* (Papilionoideae: Fabaceae): Comparative and Phylogenetic Analysis. Genes.

[39] Jia X., Wei J., Chen Y., Zeng C., Deng C., Zeng P., Tang Y., Zhou Q., Huang Y., Zhu Q. (2025). Codon Usage Patterns and Genomic Variation Analysis of Chloroplast Genomes Provides New Insights into the Evolution of Aroideae. Sci. Rep..

[40] Group T.A.P. (2016). An Update of the Angiosperm Phylogeny Group Classification for the Orders and Families of Flowering Plants: APG IV. Bot. J. Linn. Soc..

[41] Xi Z., Ruhfel B.R., Schaefer H., Amorim A.M., Sugumaran M., Wurdack K.J., Endress P.K., Matthews M.L., Stevens P.F., Mathews S. (2012). Phylogenomics and a Posteriori Data Partitioning Resolve the Cretaceous Angiosperm Radiation Malpighiales. Proc. Natl. Acad. Sci. USA.

[42] Amiryousefi A., Hyvönen J., Poczai P. (2018). IRscope: An Online Program to Visualize the Junction Sites of Chloroplast Genomes. Bioinformatics.

[43] Frazer K.A., Pachter L., Poliakov A., Rubin E.M., Dubchak I. (2004). VISTA: Computational Tools for Comparative Genomics. Nucleic Acids Res..

[44] Katoh K., Standley D.M. (2013). MAFFT Multiple Sequence Alignment Software Version 7: Improvements in Performance and Usability. Mol. Biol. Evol..

[45] Rozas J., Ferrer-Mata A., Sánchez-DelBarrio J.C., Guirao-Rico S., Librado P., Ramos-Onsins S.E., Sánchez-Gracia A. (2017). DnaSP 6: DNA Sequence Polymorphism Analysis of Large Data Sets. Mol. Biol. Evol..

[46] Yang Z. (2007). PAML 4: Phylogenetic Analysis by Maximum Likelihood. Mol. Biol. Evol..

[47] Stamatakis A. (2014). RAxML Version 8: A Tool for Phylogenetic Analysis and Post-Analysis of Large Phylogenies. Bioinformatics.

[48] Kumar S., Stecher G., Suleski M., Hedges S.B. (2017). TimeTree: A Resource for Timelines, Timetrees, and Divergence Times. Mol. Biol. Evol..

[49] Rambaut A., Drummond A.J., Xie D., Baele G., Suchard M.A. (2018). Posterior Summarisation in Bayesian Phylogenetics Using Tracer 1.7. Syst. Biol..

[50] Rambaut A. (2009). FigTree.

[51] Moore M.J., Soltis P.S., Bell C.D., Burleigh J.G., Soltis D.E. (2010). Phylogenetic Analysis of 83 Plastid Genes Further Resolves the Early Diversification of Eudicots. Proc. Natl. Acad. Sci. USA.

[52] Li X., Zhao Y., Tu X., Li C., Zhu Y., Zhong H., Liu Z., Wu S., Zhai J. (2021). Comparative Analysis of Plastomes in Oxalidaceae: Phylogenetic Relationships and Potential Molecular Markers. Plant Divers..

[53] Yao J., Zheng Z., Xu T., Wang D., Pu J., Zhang Y., Zha L. (2025). Chloroplast Genome Sequencing and Comparative Analysis of Six Medicinal Plants of *Polygonatum*. Ecol. Evol..

[54] Yang Y., Liu X., He L., Li Z., Yuan B., Fang F., Wang M., Li A., Liu C., He M. (2025). Comparative Chloroplast Genomics and Codon Usage Bias Analysis in *Hevea* Genus. Genes.

[55] Zhang J., Feng M. (2025). Analysis of the Codon Usage Bias Pattern in the Chloroplast Genomes of *Chloranthus* Species (Chloranthaceae). Genes.

[56] Yan K., Lu X., Li W., Sun C., Zhou X., Wang Y. (2025). Chloroplast Genome Diversity and Molecular Evolution in Hypericaceae: New Insights from Three *Hypericum* Species. Int. J. Mol. Sci..

[57] Zhou J., Zhang S., Wang J., Shen H., Ai B., Gao W., Zhang C., Fei Q., Yuan D., Wu Z. (2021). Chloroplast Genomes in *Populus* (Salicaceae): Comparisons from an Intensively Sampled Genus Reveal Dynamic Patterns of Evolution. Sci. Rep..

[58] Shi Y., Huang J., Wan X., Shi J., Chen Z., Zeng W. (2025). The Population Chloroplast Genomes of Populus Reveal the Phylogenetic Relationship between Three New Taxa of *Sect.* Leucoides and Their Parents. BMC Genom..

[59] Yan K., Ran J., Bao S., Li Y., Islam R., Zhang N., Zhao W., Ma Y., Sun C. (2023). The Complete Chloroplast Genome Sequence of *Eupatorium Fortunei*: Genome Organization and Comparison with Related Species. Genes.

[60] Zhong Y., Bai B., Sun Y., Wen K., Qiao Y., Guo L., Deng H., Ye Y., Feng L., Feng X. (2024). Comparative Genomics and Phylogenetic Analysis of Six Malvaceae Species Based on Chloroplast Genomes. BMC Plant Biol..

[61] Li L., Dong T., Wu D., Shu Z. (2024). The Chloroplast Genome of *Cephalanthera Nanchuanica* (Orchidaceae): Comparative and Phylogenetic Analysis with Other Neottieae Species. BMC Genom..

[62] Kim S., Park B.K., Kim H. (2024). Comparison of the Complete Chloroplast Genomes of *Astilbe*: Two Korean Endemic Plant Species. Genes.

[63] Almeida-Silva M.A., Braga-Ferreira R.S., Targueta C.P., Corvalán L.C.J., Silva-Neto C.M., Franceschinelli E.V., Sobreiro M.B., Nunes R., Telles M.P.C. (2024). Chloroplast Genomes of Simarouba Aubl., Molecular Evolution and Comparative Analyses within Sapindales. Sci. Rep..

[64] Zeng C., Hollingsworth P.M., Yang J., He Z., Zhang Z., Li D., Yang J. (2018). Genome Skimming Herbarium Specimens for DNA Barcoding and Phylogenomics. Plant Methods.

[65] Bi Y., Zhang M., Xue J., Dong R., Du Y., Zhang X. (2018). Chloroplast Genomic Resources for Phylogeny and DNA Barcoding: A Case Study on *Fritillaria*. Sci. Rep..

[66] Yu J., Han Y., Xu H., Han S., Li X., Niu Y., Chen S., Zhang F. (2023). Structural Divergence and Phylogenetic Relationships of *Ajania* (Asteraceae) from Plastomes and ETS. BMC Genom..

[67] Xiao-Ming Z., Junrui W., Li F., Sha L., Hongbo P., Lan Q., Jing L., Yan S., Weihua Q., Lifang Z. (2017). Inferring the Evolutionary Mechanism of the Chloroplast Genome Size by Comparing Whole-Chloroplast Genome Sequences in Seed Plants. Sci. Rep..

[68] Guo Y., Yang J., Bai M., Zhang G., Liu Z. (2021). The Chloroplast Genome Evolution of *Venus Slipper* (Paphiopedilum): IR Expansion, SSC Contraction, and Highly Rearranged SSC Regions. BMC Plant Biol..

[69] Shinozaki K., Ohme M., Tanaka M., Wakasugi T., Hayashida N., Matsubayashi T., Zaita N., Chunwongse J., Obokata J., Yamaguchi-Shinozaki K. (1986). The Complete Nucleotide Sequence of the *Tobacco* Chloroplast Genome: Its Gene Organization and Expression. EMBO J..

[70] Gantt J., Baldauf S., Calie P., Weeden N., Palmer J. (1991). Transfer of *Rpl22* to the Nucleus Greatly Preceded Its Loss from the Chloroplast and Involved the Gain of an Intron. EMBO J..

[71] Nakai M. (2015). YCF1: A Green TIC: Response to the de Vries et al. Commentary. Plant Cell.

[72] Dong W., Xu C., Li C., Sun J., Zuo Y., Shi S., Cheng T., Guo J., Zhou S. (2015). *Ycf1*, the Most Promising Plastid DNA Barcode of Land Plants. Sci. Rep..

[73] Souza M., Kawakita K., Slusarski S., Pereira G. (2009). Vascular Flora of the Upper Paraná River Floodplain. Braz. J. Biol..

[74] Cavallari M., Billot C., Bouvet J., Favreau B., Zucchi M.I., Palmieri D., Gimenes M. (2008). Isolation and Characterization of Microsatellite Markers for *Casearia Sylvestris Sw.* (Salicaceae), a Neotropical Medicinal Tree. Mol. Ecol. Resour..

[75] Ogutcen E., de Lima Ferreira P., Wagner N., Marinček P., Vir Leong J., Aubona G., Cavender-Bares J., Michálek J., Schroeder L., Sedio B. (2024). Phylogenetic Insights into the Salicaceae: The Evolution of Willows and Beyond. Mol. Phylogenet Evol..

[76] Dong W., Xu C., Wu P., Cheng T., Yu J., Zhou S., Hong D. (2018). Resolving the Systematic Positions of Enigmatic Taxa: Manipulating the Chloroplast Genome Data of Saxifragales. Mol. Phylogenet Evol..

