# Supplementary material for: Complete Chloroplast Genome Analysis of Casearia kurzii: Gene Loss at the IR Boundary and Monophyletic Evolution Within Casearia
Source: Plants (Basel). 2025 Apr 30;14(9):1356. doi: 10.3390/plants14091356 (PMC12073409; doi:10.3390/plants14091356)
Supplement: Supplementary file 1 [file plants-14-01356-s001.zip › Supplementary material/Table S4 Genbank accession information for 14 species.docx]

| **Species Names** | **Genbank Accession** | **Family** |
| --- | --- | --- |
| *Casearia glomerata* | NC_059787.1 | Salicaceae |
| *Casearia velutina* | MN078141.1 | Salicaceae |
| *Casearia decandra* | MN078142.1 | Salicaceae |
| *Scolopia saeva* | MN078143.1 | Salicaceae |
| *Scolopia chinensis* | MN078144.1 | Salicaceae |
| *Dovyalis caffra* | MN078137.1 | Salicaceae |
| *Abatia parviflora* | MN078139.1 | Salicaceae |
| *Bennettiodendron leprosipes* | MK301202.1 | Salicaceae |
| *Dianyuea turbinata* | NC_054283.1 | Salicaceae |
| *Flacourtia rukam* | NC_045859.1 | Salicaceae |
| *Homalium cochinchinense* | NC_045919.1 | Salicaceae |
| *Aleurites moluccanus* | MW322810.1 | Euphorbiaceae |
| *Euphorbia kansuensis* | MZ962400.1 | Euphorbiaceae |
| *Plukenetia volubilis* | NC_058006.1 | Euphorbiaceae |

**Table 1** Genbank accession information for 14 species
